# Prognostic significance of EGFR-1 and VEGF-A expression in feline cutaneous squamous cell carcinoma

**DOI:** 10.3389/fvets.2026.1949867

**Published:** 2026-10-13

**Authors:** Laís Calazans Menescal Linhares, Marcelo Monte Mor Rangel, Krishna Duro de Oliveira, Carlos Eduardo Fonseca-Alves, Fernanda Barthelson Carvalho de Moura, Guilherme Andraus Bispo, Felisbina L. Queiroga, Andrigo Barboza de Nardi

**Affiliations:** 1 Department of Veterinary Clinic and Surgery, School of Agricultural and Veterinary Sciences, São Paulo State University, Jaboticabal, São Paulo, Brazil; 2 Vet Cancer Veterinary Oncology, Brooklin, São Paulo, Brazil; 3 VCLab Pathology, Pinheiros, São Paulo, Brazil; 4 Department of Veterinary Surgery and Animal Reproduction, São Paulo State University–UNESP, Botucatu, São Paulo, Brazil; 5 School of Veterinary Medicine, São Paulo State University–UNESP, Araçatuba, São Paulo, Brazil; 6 Animal and Veterinary Science Research Center (CECAV), University of Trás-os-Montes and Alto Douro, Vila Real, Portugal

**Keywords:** EGFR-1, electrochemotherapy, cat, immunohistochemistry, prognosis, skin cancer, VEGF-A

## Abstract

**Introduction:**

Cutaneous squamous cell carcinoma (cSCC) is a prevalent malignancy in cats, and advanced cases remain challenging to manage due to their high potential for local invasion and recurrence. Epidermal growth factor receptor 1 (EGFR-1) and vascular endothelial growth factor A (VEGF-A) have emerged as promis- ing prognostic biomarkers in epithelial tumors, given their roles in proliferation, invasion, and angiogenesis. This study aimed to evaluate EGFR-1 and VEGF-A immunoexpression in cats with cSCC treated with electrochemotherapy (ECT) and their associations with clinicopathological variables and survival outcomes.

**Methods:**

Immunohistochemical expression of EGFR-1 and VEGF-A was evaluated in tumor samples from 33 cats with cSCC enrolled prospectively (n = 24) and retrospec- tively (n = 9). Associations between immunoexpression scores and clinicopatho- logical variables, overall survival time (OST), disease-free interval (DFI), and time to progression (TTP) were assessed using appropriate statistical tests.

**Results:**

EGFR-1 positivity was detected in 78.8% (26/33) of cases and was significantly associated with shorter OST, perineural invasion, and intense inflammatory infiltrate. VEGF-A immunoexpression was detected in 71.9% (23/32) of cases and was significantly associated with previous history of cSCC, perineural invasion (PNI), and tumor invasion beyond the subcutaneous fat.

**Discussion:**

These findings suggest that EGFR-1 and VEGF-A immunoexpression are associated with clinicopathological variables linked to aggressive tumor behavior in feline cSCC, supporting their potential role as prognostic biomarkers in this species and warranting further investigation in larger cohorts.

## 1 Introduction

Cutaneous squamous cell carcinoma (cSCC) is one of the most prevalent skin tumors in cats, representing a significant health concern, particularly in individuals with poorly pigmented skin and chronic exposure to ultraviolet radiation (UVR) (1). Although most cSCCs are considered curable, a subset of cats develops a more aggressive form of the disease, with high potential for local invasion and recurrence (2, 3). The early identification of patients at greater risk for adverse outcomes is imperative for better therapeutic decision-making and treatment planning (Reference).

In this context, the identification of reliable prognostic biomarkers has become a key objective in veterinary oncology, and molecular markers such as the epidermal growth factor receptor 1 (EGFR-1) and vascular endothelial growth factor A (VEGF-A) have emerged as promising candidates, given their well-established roles in tumor proliferation, invasion, and angiogenesis (4–7). Interestingly, these two molecules have been shown to act synergistically through autocrine signaling in epithelial tumor cells, promoting skin carcinoma development (8), which further supports the rationale for their simultaneous evaluation as prognostic biomarkers in feline cSCC.

EGFR-1, a transmembrane tyrosine kinase receptor, has emerged as a potential biomarker of tumor progression across multiple malignancies, receiving increasing attention in human and veterinary medicine. EGFR expression has been linked to tumor progression and more aggressive phenotypes in human cSCC (9, 10) and significantly associated with poorer survival outcomes (11). Cetuximab, a monoclonal anti-EGFR antibody, has been evaluated in advanced unresectable human cSCC, with disease control rates of 69 and 87% at 6 weeks reported in two clinical studies (10, 12). In feline cSCC, a pioneering study demonstrated a significant association between EGFR overexpression and shorter survival time (13). However, further studies are still warranted to better elucidate the role of EGFR-1 as a prognostic biomarker and therapeutic target in cats with cSCC.

The VEGF-A family is considered the most important regulator of proliferation, migration, and survival of both blood and lymphatic vasculature, with VEGF-A standing out for its pivotal role in inducing angiogenesis in pathological condition, including cancer. In canine and feline cSCC, VEGF immunoexpression has been detected in neoplastic keratinocytes but absent or at lower levels in normal skin, and its expression has been significantly associated with disease progression and recurrence, supporting a potential role of VEGF-driven angiogenesis in the biological behavior of these tumors (6, 14).

In humans, the prognostic impact of VEGF-A expression has been more extensively evaluated in mucosal head and neck SCC, where high VEGF-A expression has been associated with advanced disease and worse prognosis (15, 16). In cSCC specifically, high VEGF-A expression has been reported in association with poorly differentiated tumors and more aggressive phenotypes (17, 18). Regarding targeted therapy, Bevacizumab, a monoclonal antibody targeting VEGF-A, has been evaluated in recurrent and metastatic mucosal head and neck SCC. Although a phase III clinical trial demonstrated significant improvements in response rate and progression-free survival, no benefit in overall survival was observed, and high toxicity rates were reported (19).

Considering the biological and histopathological similarities between feline and human cSCC, including shared etiological factors, biological behavior, and molecular pathways, the cat has been proposed as a valuable spontaneous animal model for the study of human cSCC (20). In this context, a better understanding of the prognostic role of EGFR-1 and VEGF-A in feline cSCC may not only improve clinical management in veterinary oncology, but also contribute to the development of targeted therapies applicable to both species.

Electrochemotherapy (ECT) has emerged as an effective local treatment modality for feline and human cSCC, particularly for tumors in anatomical locations where achieving adequate surgical margins may be challenging. ECT combines the administration of a cytotoxic drug, most commonly bleomycin, with the delivery of short, high-intensity electric pulses that transiently increase tumor cell membrane permeability, thereby enhancing intracellular drug uptake and cytotoxicity (21). In addition to this direct cytotoxic effect, ECT induces vascular effects characterized by transient vasoconstriction and disruption of tumor blood vessels, which may contribute to tumor control by reducing blood flow and enhancing local drug retention (22). High objective response rates have been reported in humans and cats with cSCC treated with ECT, ranging from 83 to 100% (2, 3, 21, 23), supporting its clinical relevance as a local treatment for this disease.

Therefore, this study aimed to evaluate the immunoexpression of EGFR-1 and VEGF-A in a cohort of cats with cSCC treated with ECT, and to investigate their potential associations with clinicopathological variables and survival outcomes. By identifying clinically relevant biomarkers shared between feline and human cSCC, this study also aims to contribute to the comparative oncology framework, further supporting the cat as a spontaneous translational model for the investigation of targeted therapeutic strategies.

## 2 Materials and methods

### 2.1 Study design and case selection

This study included cats with histologically confirmed cutaneous squamous cell carcinoma (cSCC) located in the head region and treated with ECT. Cats were prospectively enrolled from the Veterinary Oncology Service of the Veterinary Teaching Hospital, São Paulo State University (UNESP), Jaboticabal, Brazil, and from the VetCancer Care Oncology Center, São Paulo, Brazil, between January 2023 and July 2025. Additionally, medical records from the VetCancer Care Oncology Center were retrospectively reviewed to identify cats with cSCC treated with ECT between January 2018 and December 2022.

The study was approved by the Animal Ethics Committee of Universidade Estadual Paulista (UNESP/FCAV; protocol no. 7726/22) and conducted in accordance with the National Council for the Control of Animal Experimentation (CONCEA) guidelines. Cats were eligible for inclusion if they met the following criteria: histopathological confirmation of cSCC, indication for ECT as the sole therapeutic modality, and no evidence of distant metastatic disease at initial presentation. Exclusion criteria included any condition contraindicating general anesthesia. Cats with a follow-up period of less than 30 days after treatment were excluded from survival analyses.

Clinical data were obtained from both the prospective and retrospective cohorts. In the prospective cohort, cats underwent standardized clinical evaluation, staging, treatment, and follow-up protocols. In the retrospective cohort, equivalent clinical and outcome data were retrieved from medical records. Data recorded for all cats included: demographic information (age, sex, and breed), history of previous cSCC, tumor characteristics (size, location, number of lesions, growth pattern, and lip involvement), clinical staging workup (thoracic radiography and regional lymph node evaluation), tumor stage according to the World Health Organization (WHO) classification (24), ECT treatment details, tumor response to ECT, and outcome data.

### 2.2 Treatment protocol

All cats underwent ECT under general anesthesia with perioperative analgesia. Bleomycin (Bonar®, Aché, Brazil) was diluted in saline solution and administered intravenously as a bolus at a dose of 15,000 IU/m^2^. Eight minutes after bleomycin administration, electric pulses were delivered to the tumor and surrounding healthy tissue margins (1–2 cm) using needle electrodes. Each pulse train consisted of eight pulses at an electric field intensity of 1,000 V/cm, a frequency of 5 kHz, and a pulse duration of 100 μs. The same electroporation protocol was used in both cohorts, differing only in the pulse generator used (E-pore® for prospective cases and VETCP 125® for retrospective cases).

After treatment, clinical reassessments were performed at 7-day intervals during the first month after ECT and every 15 days thereafter. Tumor response was assessed according to RECIST criteria (25) and categorized as complete response (CR), partial response (PR), stable disease (SD), or progressive disease (PD). Response classification was performed from 30 days post-treatment onward, after adequate tissue healing.

### 2.3 Histopathology

Tumor samples were obtained by incisional biopsy prior to ECT initiation. All samples were fixed in 10% formalin, routinely processed, and embedded in paraffin. Histological sections were stained with hematoxylin and eosin (H&E) and evaluated by the same board-certified veterinary pathologist. In retrospective cases, archived histological slides were reviewed by the same pathologist to ensure consistency in diagnosis and histological grading.

The standard histological features assessed included histological classification (well-differentiated, moderately differentiated, poorly differentiated, acantholytic, and *in situ*) according to Goldschmidt and Goldschmidt (26), intensity of inflammatory infiltrate (mild, moderate, or intense), and mitotic index (MI). MI was determined as the total number of mitotic figures counted in a standardized area of 2.37 mm^2^ (10 high-power fields at 400 × magnification). Additional histological parameters were assessed based on criteria previously described for human cSCC, including depth of invasion (superficial, intermediate, and deep), invasion beyond the subcutaneous fat (present or absent), and perineural invasion (PNI; present or absent) (2, 11, 27).

### 2.4 Immunohistochemistry

The clone of both anti-EGFR-1 (28) and anti-VEGF-A (29) antibodies have been previously validated for cross-reactivity with feline tissues in immunohistochemical studies. The immunohistochemical analyses were performed to evaluate the expression of EGFR-1 and VEGF-A in tumor samples following standardized protocols. Sections of 4 μm thickness were obtained from formalin-fixed paraffin-embedded (FFPE) tumor tissues and mounted on positively charged slides (StarFrost, Knittel, Bielefeld, Germany). Deparaffinization was performed using xylene, followed by antigen retrieval in a high-pH buffer solution (Dako, Carpinteria, CA, United States) using a Pascal pressure chamber (Dako) for 45 min. Endogenous peroxidase activity was blocked by incubation with hydrogen peroxide for 15 min at room temperature. Non-specific protein binding was blocked using skimmed milk (Molico®, Nestlé, Brazil) diluted in distilled water for 40 min at 27 °C in a humidified chamber. Sections were then incubated overnight at 4 °C with the primary antibodies anti-EGFR1 (clone Ab-5/31G7, mouse monoclonal, Thermo Fisher Scientific, United States) and anti-VEGFA (mouse monoclonal, clone VG1, Dako, Unites States), both at a dilution of 1:100 (13, 30). Following primary antibody incubation, slides were washed in phosphate-buffered saline (PBS), and immunostaining was developed using the EnVision™ FLEX/HRP detection system (Dako, Carpinteria, CA, United States) with 3,3′-diaminobenzidine (DAB) as chromogen. Sections were counterstained with hematoxylin, dehydrated, cleared, and coverslipped. Skin samples were used as positive controls for both EGFR1 and the blood cells located in dermis were control for VEGFA, selected based on expression profiles reported in the Human Protein Atlas (31). Negative controls were performed by omitting the primary antibody.

### 2.5 Immunohistochemical evaluation

Immunohistochemical slides were examined under a light microscope (26 mm diameter; Leica DMLB, HC PLAN 10×/20) at 400 × magnification. As the samples consisted of small incisional biopsies collected before ECT, the available viable tumor tissue was evaluated throughout the section at 400 × magnification, rather than using a predetermined number of high-power fields or a predefined minimum number of neoplastic cells. Areas of necrosis and tissue-processing artifacts were excluded from the assessment. Immunohistochemical scoring was performed by two observers blinded to clinical outcomes, and the final score was assigned by consensus.

EGFR-1 and VEGF-A expression was evaluated in tumor epithelial cells according to staining localization (EGFR-1: membranous and cytoplasmic; VEGF-A: predominantly cytoplasmic). Immunoreactivity scores were calculated semi-quantitatively based on staining intensity and the percentage of positive tumor cells.

For EGFR-1, the percentage of positive tumor cells was scored as 1 (≤10%), 2 (11–30%), 3 (31–60%), or 4 (>60%). Staining intensity was graded as 1, low; 2, moderate; or 3, high. The immunoreactivity score was calculated as the product of the percentage and intensity scores. Tumors with scores ≤2 were classified as negative, whereas positive tumors were further classified as having low (3–5), moderate (6–8), or high (9–12) expression, according to previously published criteria (13).

For VEGF-A, a scoring system adapted from previously published methods (4, 29) was used. The percentage of positive tumor cells was scored as 1 (<10%), 2 (10–25%), 3 (26–50%), or 4 (>50%), while staining intensity was graded as 0 (no staining), 1 (weak), 2 (moderate), or 3 (strong). The final immunoreactivity score was calculated as the sum of the percentage and intensity scores, resulting in a total score ranging from 1 to 7. Tumors were classified as negative (1–3), weakly positive (4, 5), or strongly positive (6, 7).

### 2.6 Statistical analysis

Statistical analyses were performed using IBM SPSS Statistics version 31.0 (IBM Corp., Armonk, NY, United States). Clinicopathological and ECT-related characteristics were summarized using absolute and relative frequencies. Associations between categorical variables were assessed using the chi-square test or Fisher’s exact test, as appropriate. The correlation between EGFR-1 and VEGF-A immunoreactivity scores was assessed using Spearman’s rank correlation coefficient.

Survival outcomes were estimated using the Kaplan–Meier method and compared between groups using the log-rank test. Disease-free interval (DFI) was calculated from the date of the first ECT session to the date of local recurrence, and was computed exclusively for cats that achieved complete response. Time to progression (TTP) was measured from the first ECT session to evidence of tumor progression, and was computed for cats that achieved complete response or partial response. Overall survival time (OST) corresponded to the interval between the first ECT session and death attributable to cSCC. For all time-to-event endpoints, cats that were lost to follow-up, died from tumor-unrelated causes, or remained alive without evidence of recurrence or progression at study completion were censored at the time of last contact. Statistical significance was set at *p* < 0.05.

## 3 Results

### 3.1 Baseline characteristics of the study cohort

A total of 33 cats were included in the study, comprising 24 (72.7%) from the prospective cohort and 9 (27.3%) from the retrospective cohort. One target lesion per cat was selected for analysis based on clinical relevance, resulting in 33 tumor samples available for immunohistochemical evaluation. The distribution of demographic, clinical, histologic and ECT-related variables are summarized in Table 1.

**Table 1:** Demographic, clinical, histological and treatment-related variables of 33 cats with cutaneous squamous cell carcinoma

Demographic and clinical variables		*n* (%)
Sex (*n* = 33)	Female	20 (60.6)
	Male	13 (39.4)
Age (*n* = 33)	Young adult (1–6 years)	6 (18.2)
	Mature adult (7–10 years)	11 (33.3)
	Senior (≥11 years)	16 (48.5)
Coat color (*n* = 33)	White	10 (30.3)
	Partially white	23 (69.7)
Number of lesions (*n* = 33)	Single	25 (75.8)
	Multiple	8 (24.2)
Anatomic location (*n* = 33)	Nasal planum	17 (51.5)
	Eyelid	4 (12.1)
	Ear	5 (15.2)
	Temporal	3 (9.1)
	Labial	2 (6.1)
	Mental	2 (6.1)
Tumor size (cm) (*n* = 28)	≤2.5	19 (67.9)
	>2.5	9 (32.1)
Labial extension (*n* = 33)	No	18 (54.5)
	Yes	15 (45.5)
Previous cSCC (*n* = 33)	No	24 (72.7)
	Yes	9 (27.3)
Growth pattern (*n* = 33)	Non-erosive	15 (45.5)
	Erosive	18 (54.5)
T stage (*n* = 33)	T1	8 (24.2)
	T2	10 (30.3)
	T3	3 (9.1)
	T4	12 (36.4)
Histological variables		
Histopathological classification (*n* = 33)	Well-differentiated	6 (18.2)
	Moderate differentiated	8 (24.2)
	Poorly differentiated	13 (39.4)
	Acantholytic	5 (15.2)
	*In situ*	1 (3.0)
Mitotic index (*n* = 32)	<7	15 (46.9)
	≥7	17 (53.1)
Depth of invasion (*n* = 30)	Superficial (≤2 mm)	9 (30)
	Intermediate (3–5 mm)	14 (46.7)
	Deep (>5 mm)	7 (23.3)
Invasion beyond subcutaneous fat (*n* = 25)	No	17 (68)
	Yes	8 (32)
Inflammatory intensity (*n* = 32)	Mild	4 (12.5)
	Moderate	15 (46.9)
	Intense	13 (40.6)
Perineural invasion (*n* = 24)	No	12 (50)
	Yes	12 (50)
ECT-related variables		
Tumor response (*n* = 30)	CR	24 (80%)
	PR	6 (20%)
Local recurrence (*n* = 24)	No	15 (62.5)
	Yes	9 (37.5)

cSCC, cutaneous squamous cell carcinoma; ECT, electrochemotherapy; CR, complete response; PR, partial response.

### 3.2 Response to electrochemotherapy

Clinical response following ECT was evaluable in 30 of 33 cats (90.9%); three cats were excluded because they did not complete the minimum 30-day follow-up period required for response assessment. The best response achieved during the treatment course was CR in 24 cats (80.0%) and PR in 6 cats (20.0%). Among the 24 cats that achieved CR, 20 (83.3%) achieved CR after a single ECT session, whereas 4 (16.7%) achieved CR after a second ECT session, having shown an initial tumor reduction of ≥90% after the first treatment.

### 3.3 EGFR-1 and VEGF-A immunoexpression

EGFR-1 positivity was detected in 26 of 33 samples (78.8%), while 7 cases (21.2%) were classified as negative. (Figure 1A) All positive samples (immunoractivity score > 2) exhibited predominantly cytoplasmic EGFR-1 immunoreactivity (Figures 1B,C). Immunoreactivity scores ranged from 0 to 9, with a median score of 6. Among positive cases, 10 (38.5%) showed low-level expression (scores 3–5), 15 (57.7%) showed moderate expression (scores 6–8), and 1 (3.8%) showed high expression (score 9), according to the classification proposed by Sabattini et al. (13).

**Figure 1:**
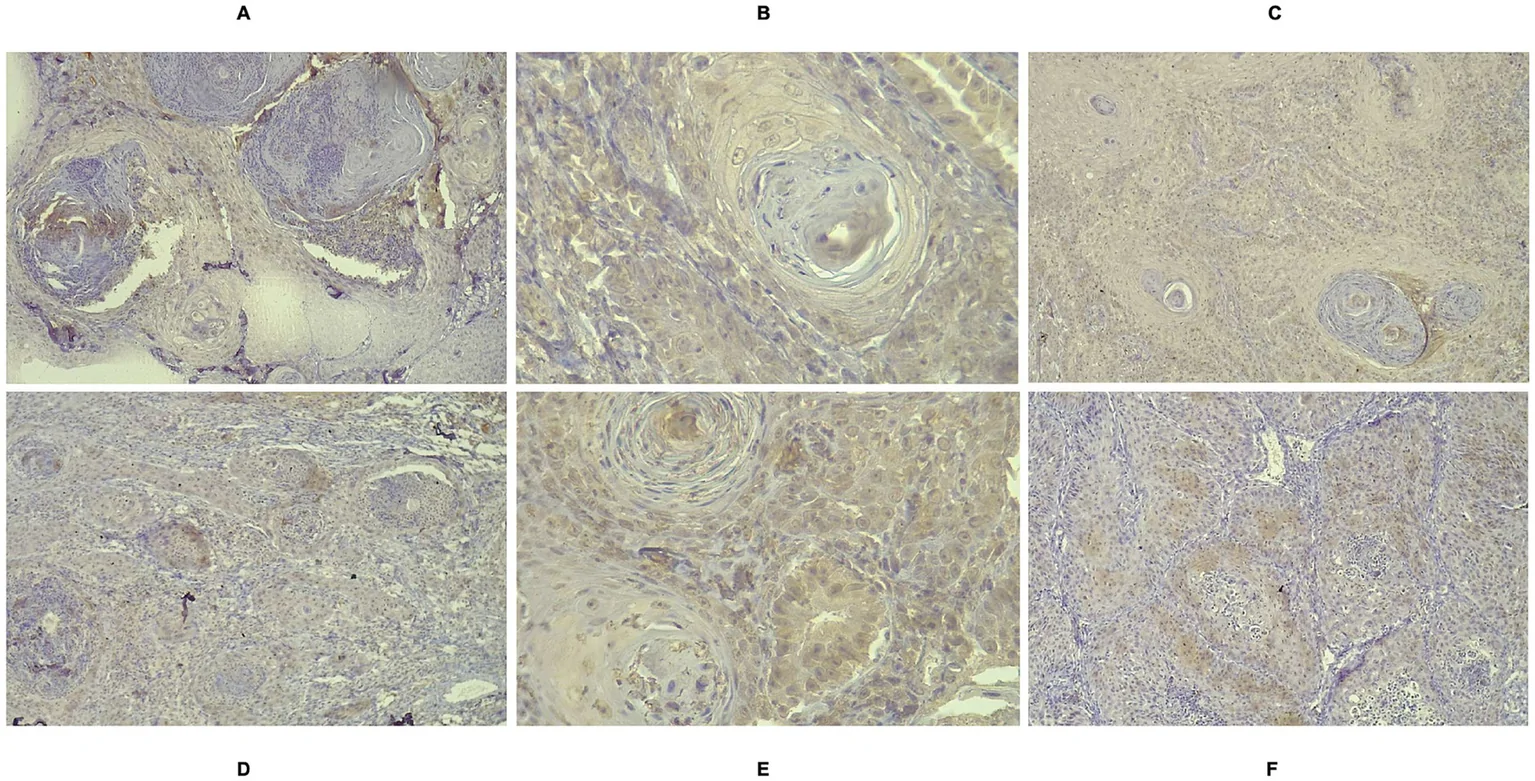
Representative photomicrographs of EGFR-1 and VEGF-A immunoreactivity in feline cutaneous squamous cell carcinoma. **(A)** Poorly differentiated cSCC of the eyelid showing weak EGFR immunoreactivity (1+) in a minority of neoplastic epithelial cells (score 2; 11–30%), resulting in a negative immunoreactivity score of 2. **(B)** Moderately differentiated cSCC of the nasal planum showing moderate EGFR immunoreactivity (2+) in the cytoplasm of the majority of neoplastic epithelial cells (4; >60%), resulting in a positive immunoreactivity score of 8. A keratin pearl with reduced staining in centrally located differentiated cells is observed. **(C)** Acantholytic cSCC of the ear showing moderate (2+) and diffuse (score 4; >60%) EGFR-1 immunoreactivity in the cytoplasm of neoplastic epithelial cells, resulting in a positive immunoreactivity score of 8. **(D)** Acantholytic cSCC of the ear showing moderate (2+) and diffuse (score 4; >50%) VEGF-A immunoreactivity in the cytoplasm of neoplastic epithelial cells, resulting in a strongly positive immunoreactivity score of 6. **(E)** Moderately differentiated cSCC of the nasal planum showing strong VEGF immunoreactivity (3+) in the cytoplasm of the majority of neoplastic epithelial cells (4; >50%), resulting in a strongly positive immunoreactivity score of 7. **(F)** Well-differentiated cSCC of the mental region showing moderate (2+) VEGF-A immunoreactivity in the cytoplasm of the majority of neoplastic epithelial cells (4; >50%), resulting in a strongly positive immunoreactivity score of 6. Counterstained with hematoxylin. Original magnification: **(A, B, C, F)** 100×; **(B, E)** 400 ×.

VEGF-A immunostaining was evaluable in 32 of the 33 tumor samples, as one sample could not be assessed due to tissue section detachment from the slide during immunohistochemical processing. VEGF-A immunostaining was positive in 23 of the 32 evaluable samples (71.9%), with 16 (50.0%) classified as weakly positive (scores 4–5) and 7 (21.9%) as strongly positive (scores 6–7; Figures 1D–F). Nine samples (28.1%) were classified as negative (scores 0–3). Immunoreactivity scores ranged from 0 to 7, with a median score of 4.

A significant positive correlation was observed between EGFR-1 and VEGF-A immunoreactivity scores (Spearman’s ρ = 0.692, *p* < 0.001; n = 32), indicating that higher EGFR-1 scores were associated with higher VEGF-A scores (Figure 2).

**Figure 2:**
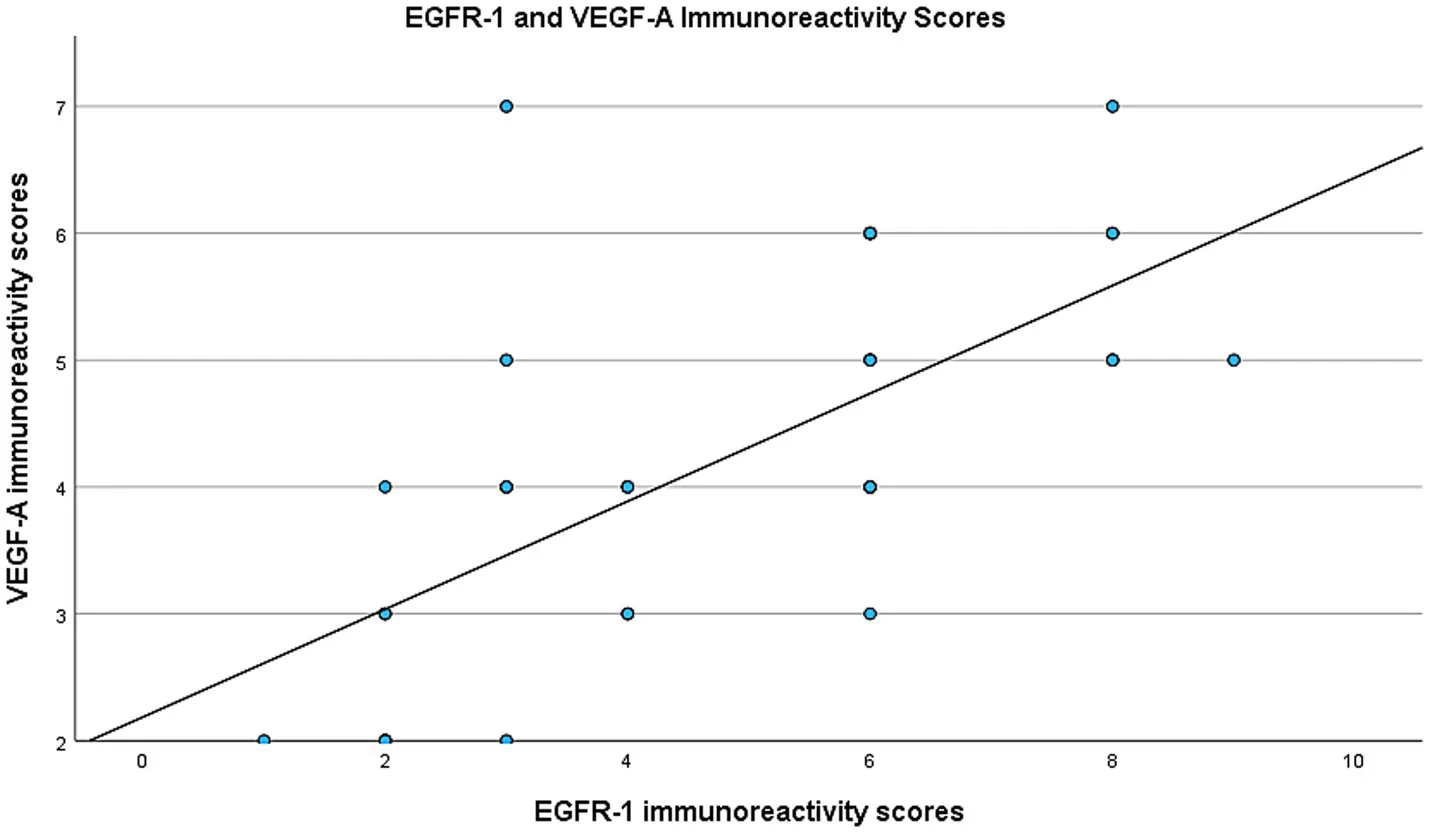
Relationship between EGFR-1 and VEGF-A immunoreactivity scores in feline cutaneous squamous cell carcinoma. A significant positive correlation was observed between EGFR-1 and VEGF-A scores (Spearman’s *ρ* = 0.692, *p* < 0.001; *n* = 32). The line represents the linear trend of the observed scores.

### 3.4 Clinicopathological associations of EGFR-1 and VEGF-A immunoexpression

The distribution of clinicopathological variables according to EGFR-1 and VEGF-A immunoreactivity is presented in Table 2. EGFR-1 immunoreactivity was significantly associated with PNI (*p* = 0.047) and intense inflammatory infiltrate (*p* = 0.045). VEGF-A expression was significantly associated with a previous history of cutaneous squamous cell carcinoma (cSCC; *p* = 0.038), PNI (*p* = 0.033), and invasion beyond the subcutaneous fat (*p* = 0.028).

**Table 2:** Association between clinicopathological parameters and EGFR and VEGF immunoreactivity in 33 feline cutaneous squamous cell carcinomas.

Clinicopathological parameters	EGFR expression		*p*-value	VEGF expression			*p*-value
	Negative (%)	Positive (%)		Negative (%)	Weakly positive (%)	Strongly positive (%)	
Sex (*n* = 33)							
F	5 (15.2)	15 (45.5)	0.676	6 (18.8)	10 (31.3)	3 (9.4)	0.590
M	2 (6.1)	11 (33.3)		3 (9.4)	6 (18.8)	4 (12.5)	
Tumor location (*n* = 33)							
Ear	2 (6.1)	3 (9.1)	0.767	2 (6.3)	2 (6.3)	1 (3.1)	0.565
Eyelid	1 (3)	3 (9.1)		0 (0)	3 (9.4)	1 (3.1)	
Labial	0 (0)	2 (6.1)		0 (0)	1 (3.1)	1 (3.1)	
Mental region	0 (0)	2 (6.1)		0 (0)	1 (3.1)	1 (3.1)	
Nasal plane	3 (9.1)	14 (42.4)		5 (15.6)	9 (28.1)	2 (6.3)	
Temporal	1 (3)	2 (6.1)		2 (6.3)	0 (0)	1 (3.1)	
Tumor size (*n* = 28)							
≤2.5 cm	4 (14.3)	15 (53.6)	1.000	4 (14.3)	12 (42.9(	3 (10.7)	0.323
>2.5 cm	1 (3.6)	8 (28.6)		3 (10.7)	3 (10.7)	3 (10.7)	
Multiplicity of lesions (*n* = 33)							
Single lesion	4 (12.1)	21 (63.6)	0.320	6 (18.8)	14 (43.8)	4 (12.5)	0.240
Multiple lesions	3 (9.1)	5 (15.2)		3 (9.4)	2 (6.3)	3 (9.4)	
Previous cSCC (*n* = 33)							
No	7 (21.2)	17 (51.5)	0.149	9 (28.1)	11 (34.4)	3 (9.4)	**0.038**
Yes	0 (0)	9 (27.3)		0 (0)	5 (15.6)	4 (12.5)	
Labial extension (*n* = 33)							
No	5 (15.2)	13 (39.4)	0.312	5 (15.6)	9 (28.1)	4 (12.5)	0.998
Yes	2 (6.1)	13 (39.4)		4 (12.5)	7 (21.9)	3 (9.4)	
Growth pattern (*n* = 33)							
Non-erosive	2 (6.1)	13 (39.4)	0.413	3 (9.4)	9 (28.1)	3 (9.4)	0.529
Erosive	5 (15.2)	13 (39.4)		6 (18.8)	7 (21.9)	4 (12.5)	
T stage (*n* = 33)							
T1	1 (3)	7 (21.2)	0.629	1 (3.1)	4 (12.5)	3 (9.4)	0.364
T2	3 (9.1)	7 (21.2)		4 (12.5)	5 (15.6)	1 (3.1)	
T3	0 (0)	3 (9.1)		0 (0)	3 (9.4)	0 (0)	
T4	3 (9.1)	9 (27.3)		4 (12.5)	4 (12.5)	3 (9.4)	
cSCC classification (*n* = 32)							
Well-differentiated	2 (6.3)	4 (12.5)	0.766	3 (9.7)	0 (0)	3 (9.7)	0.185
Moderately differentiated	1 (3.1)	7 (21.9)		1 (3.2)	6 (19.4)	1 (3.2)	
Poorly differentiated	2 (6.3)	11 (34.4)		3 (9.7)	8 (25.8)	2 (6.5)	
Acantholytic	1 (3.1)	4 (12.5)		1 (3.2)	2 (6.5)	1 (3.2)	
Mitotic index (*n* = 32)							
≤6	4 (12.5)	11 (34.4)	0.383	4 (12.9)	6 (19.4)	4 (12.9)	0.650
>6	2 (6.3)	15 (46.9)		4 (12.9)	10 (32.3)	3 (9.7)	
Depth of invasion (*n* = 30)							
Superficial (≤2 mm)	1 (3.3)	8 (26.7)	0.220	3 (10.3)	3 (10.3)	3 (10.3)	0.094
Intermediate (>2 mm–5 mm)	4 (13.3)	10 (33.3)		4 (13.8)	6 (20.7)	3 (10.3)	
Deep (>5 mm)	0 (0)	7 (23.3)		0 (0)	7 (24.1)	0 (0)	
Invasion beyond subcutaneous fat (*n* = 25)							
No	4 (16)	13 (52)	1.000	6 (25)	5 (20.8)	5 (20.8)	**0.028**
Yes	1 (4)	7 (28)		0 (0)	7 (29.2)	1 (4.2)	
Inflammatory intensity (*n* = 32)							
Mild (+)	2 (6.3)	2 (6.3)	**0.045**	2 (6.5)	2 (6.5)	0 (0)	0.193
Moderate (++)	4 (12.5)	11 (34.4)		5 (16.1)	8 (25.8)	2 (6.5)	
Intense (+++)	0 (0)	13 (40.6)		1 (3.2)	6 (19.4)	5 (16.1)	
PerineuraI invasion (*n* = 24)							
No	4 (16.7)	8 (33.3)	**0.047**	5 (21.7)	6 (26.1)	1 (4.3)	**0.033**
Yes	0 (0)	12 (50)		0 (0)	7 (30.4)	4 (17.4)	
Tumor response (*n* = 30)							
CR	6 (20)	18 (60)	1.000	7 (24.1)	10 (34.5)	7 (24.1)	0.382
PR	1 (3.3)	5 (16.7)		2 (6.9)	3 (10.3)	0 (0)	
Local recurrence (*n* = 25)							
Yes	5 (20)	11 (44)	0.364	5 (20.8)	7 (29.2)	3 (12.5)	0.443
No	1 (4)	8 (32)		2 (8.3)	3 (12.5)	4 (16.7)	

cSCC, cutaneous squamous cell carcinoma; CR, complete response; PR, partial response. Bold values indicate statistically significant associations (*p* < 0.05).

### 3.5 Survival analysis

Survival analyses included 30 cats, as three cases were excluded because follow-up was less than 30 days after treatment, according to the predefined exclusion criterion. Briefly, at the end of the study period, 13 (43.3%) cats were alive, while 17 (56.7%) had died, either due to tumor progression (11/17, 64.7%) or causes unrelated to the tumor (6/17, 35.3%). Overall median OST was 601 days (95% CI, 180–1,021). Mean DFI was 588 days (95% CI, 405–764) and median TTP was 99 days (95% CI, 73–124).

Kaplan–Meier survival analysis demonstrated that cats with EGFR-positive tumors had significantly shorter survival than those with EGFR-negative tumors (log-rank test, *p* = 0.033; Figure 3). For VEGF, Kaplan–Meier survival analysis revealed no significant association between VEGF expression and any survival outcome (*p* > 0.05).

**Figure 3:**
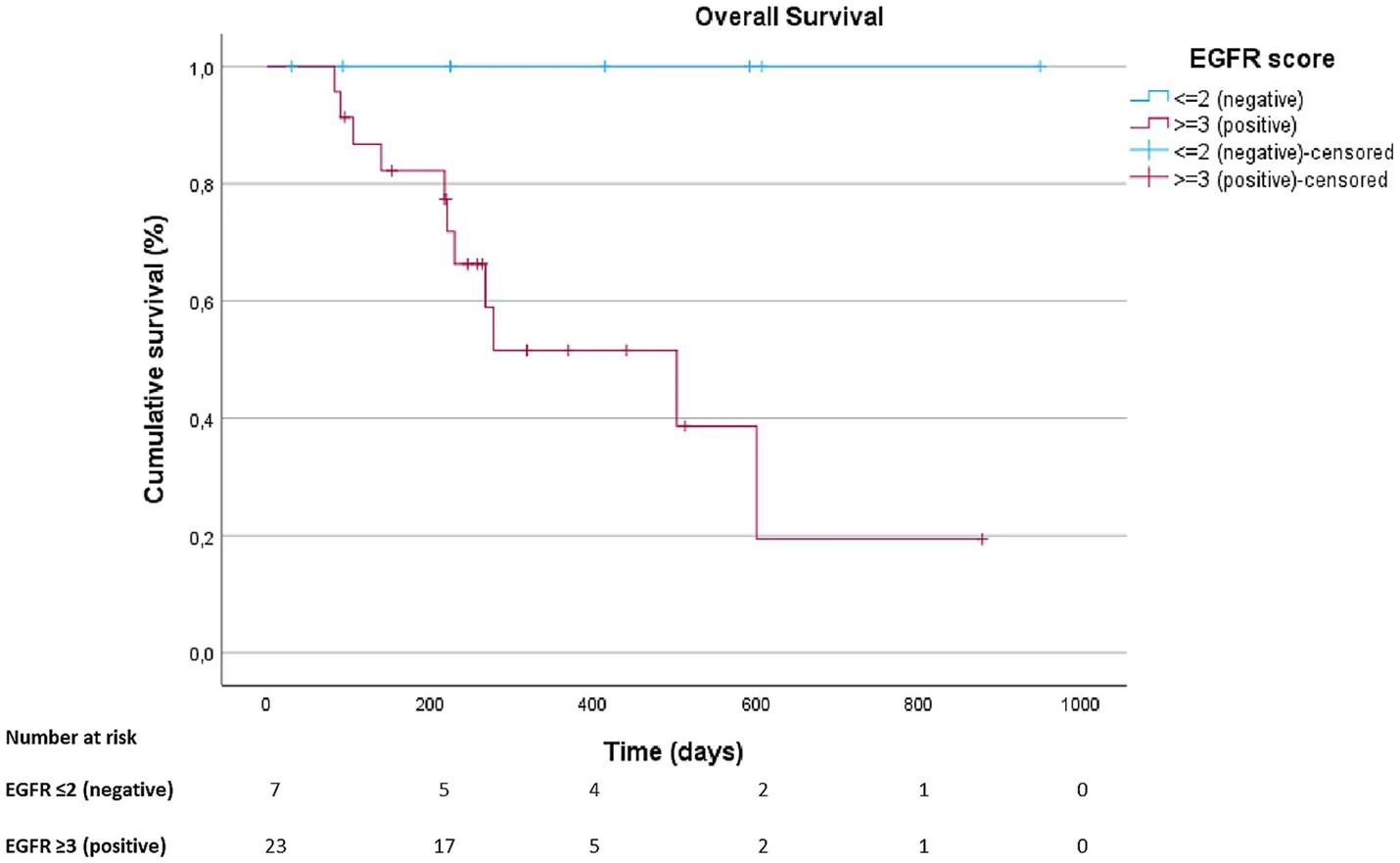
Kaplan–Meier curve comparing survival of the cats based on the EGFR immunohistochemical expression. Subjects with negative-EGFR tumors survived for a longer time than subjects positive-EGFR tumors (*p* = 0.033). The number of cats at risk at each time point is shown below the survival curve.

Additionally, PNI-positive cats showed a significantly shorter survival than those cats negative for PNI (mean OST of 327 *vs.* 789 days, respectively; *p* = 0.027, Figure 4).

**Figure 4:**
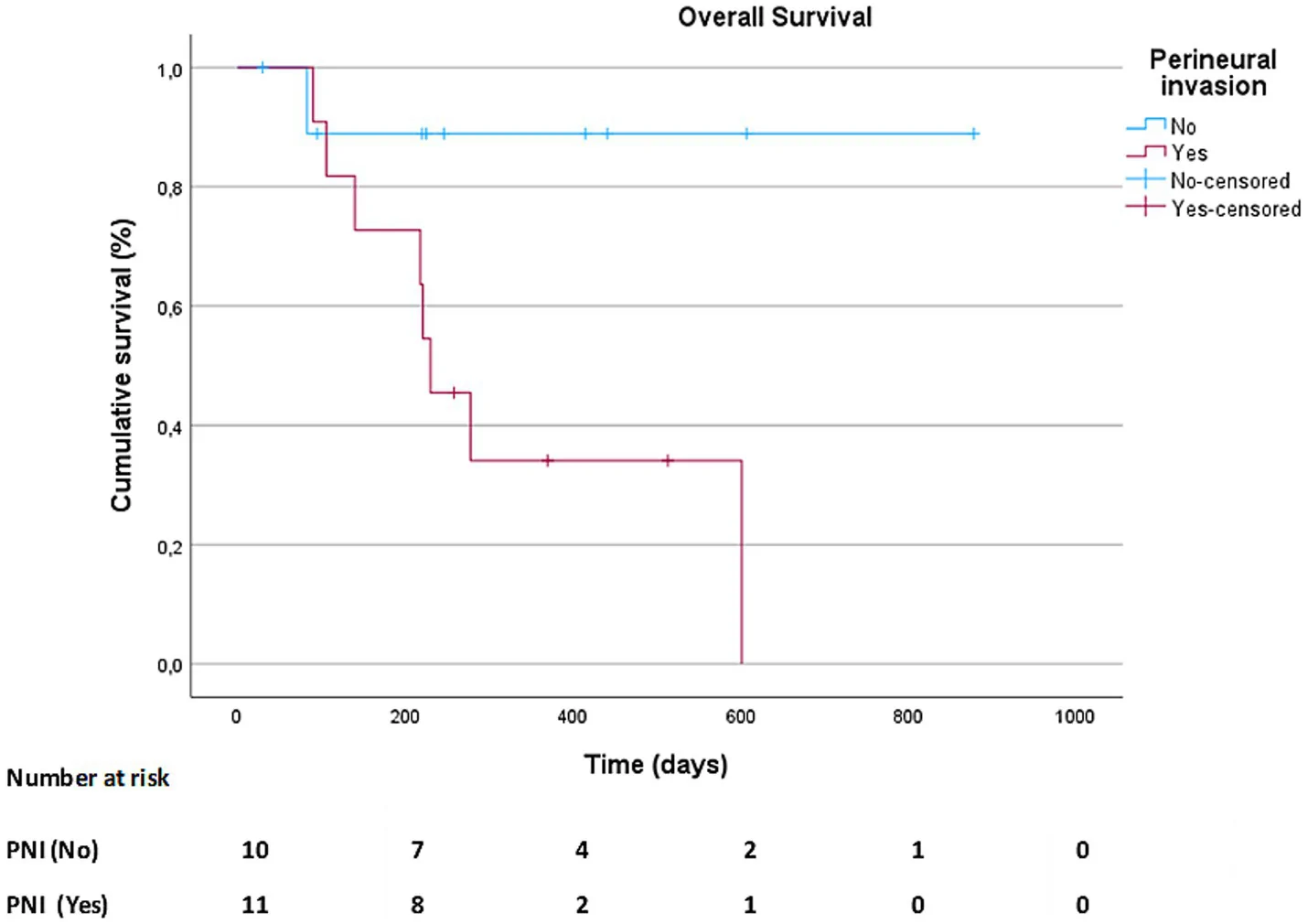
Kaplan–Meier curve comparing survival of the cats based on the presence of PNI. Subjects with PNI-negative tumors survived for a longer time than subjects PNI-negative tumors (*p* = 0.027). The number of cats at risk at each time point is shown below the survival curve.

## 4 Discussion

The identification of reliable prognostic and predictive biomarkers remains a central challenge in veterinary oncology, and studies evaluating EGFR-1 and VEGF-A expression in feline cSCC are still scarce. Therefore, this prospective-retrospective study aimed to evaluate the immunoexpression of EGFR-1 and VEGF-A in 33 cats with cSCC and their associations with clinicopathological and survival outcomes.

EGFR-1 is a transmembrane tyrosine kinase receptor that dimerizes and autophosphorylates upon ligand binding, triggering a downstream cascade of intracellular signaling pathways that ultimately stimulate the transcription of genes involved in cell proliferation, differentiation, motility, and survival. EGFR-1 may also be detected in the cytoplasm, reflecting its recycling pathway and the dysregulation of receptor processing associated with tumorigenesis (11, 32). In the present study, EGFR-1 positivity was detected in 78.8% (26/33) of cases, consistent with the positivity rate of 74% previously reported in feline cSCC (13), and with the prevalence of EGFR-1 expression reported in human cSCC (11, 33).

EGFR-1 positive immunoexpression was significantly associated with shorter OST. To date, only one published study has investigated the prognostic value of EGFR-1 immunoexpression in feline cSCC, and its findings are consistent with ours, reporting a significant association between EGFR-1 overexpression and reduced survival time (13). Therefore, our results reinforce the potential for EGFR-1 as prognostic markers for cSCC. In human cSCC, although some studies have failed to demonstrate a significant association between EGFR-1 overexpression and adverse outcomes (34, 35), more recent studies have reported significant correlations with tumor progression and metastasis (11). Furthermore, the identification of EGFR-1 overexpression in feline cSCC may also have therapeutic implications, supporting the rationale for investigating anti-EGFR targeted therapies as a potential treatment strategy for cats with EGFR-1–positive tumors.

Recently, some studies have been published on the use of HER2/EGFR inhibitors in cats with cancer (36–38). These studies provide encouraging evidence that targeting the EGFR/HER2 signaling pathway is both feasible and clinically relevant in feline oncology (36–38). In particular, lapatinib has demonstrated acceptable safety and preliminary antitumor activity in naturally occurring feline neoplasms, while preclinical investigations have shown that EGFR-1 inhibition can reduce tumor cell proliferation, enhance radiosensitivity, and potentially overcome resistance mechanisms (36–38). These findings support the rationale for further investigating EGFR-1 and HER2-targeted therapies as precision medicine approaches for cats with EGFR-expressing malignancies.

Perineural invasion (PNI) is defined as the ability of tumor cells to spread into, around, or through the surrounding nerves, and is widely recognized as a relevant prognostic indicator associated with poorer outcomes in human cSCC (39, 40). In feline cSCC, a previous retrospective study evaluating cats treated with ECT found a significant association between PNI and local recurrence; however, its potential association with survival was not investigated (2).

In the present study, EGFR-1 positivity was significantly associated with PNI. This finding is consistent with evidence from human SCC of mucosal origin, in which EGFR-1 expression was significantly associated with PNI, with the vast majority of PNI-positive cases exhibiting EGFR-1 immunoreactivity (41, 42). EGFR signaling has been implicated in PNI through pathways involving tumor-derived neuropeptides, Src kinase activation, MAPK/ERK1/2 signaling, and upregulation of matrix metalloproteinases, as well as paracrine loops between tumor cells and nerve tissue (42, 43). Although comparable data in feline cSCC are lacking, the conservation of EGFR-1 signaling pathways across species supports the biological plausibility of this association.

In the present study, EGFR-1 positivity was significantly associated with intense inflammatory infiltrate, a finding that may be explained by a bidirectional relationship between EGFR-1 signaling and the tumor inflammatory microenvironment. On one hand, EGFR-1 activation drives the expression of pro-inflammatory cytokines and chemokines — including IL-6, TNF, and IL-8 — that actively recruit inflammatory cells to the tumor microenvironment, potentially accounting for the more intense infiltrate observed in EGFR-positive tumors (43). On the other hand, inflammatory mediators may reciprocally amplify EGFR signaling, notably through substance P, which transactivates EGFR-1 via Src kinase, activates the MAPK/ERK1/2 pathway, and further upregulates MMPs and pro-inflammatory molecules (43). This self-reinforcing loop may contribute to the more aggressive biological behavior observed in EGFR-positive tumors in the present study, including the associations with PNI and reduced survival time.

Although EGFR-1 plays an important role in cell differentiation and proliferation, our results failed to demonstrate a significant association between EGFR-1 immunoexpression and mitotic activity or tumor differentiation, consistent with previous findings (13). In contrast, previous studies in humans have reported significantly higher EGFR-1 expression in moderately to poorly differentiated cutaneous and head and neck SCC (11, 44). These conflicting results may be attributable to differences in sample size and the immunohistochemical scoring systems employed across studies.

The development of new blood vessels, also called neoangiogenesis, is a crucial event for tumor development and spread (45). VEGF-A, a heparin-binding growth factor, acts directly on vascular endothelial cells, promoting cell growth, migration, and vascular permeability in physiological and pathological conditions. VEGF-A immunoexpression has been detected in neoplastic keratinocytes of canine and feline and canine cSCC but not in normal skin, and its expression has been significantly associated with disease progression and recurrence (14). Similarly, a subsequent study reported increased VEGF-A immunoexpression in feline cSCC compared to normal skin (6). These findings suggest a potential role of VEGF-driven angiogenesis in the biological behavior of these tumors.

In the present study, VEGF-A immunoexpression was detected in 71.9% of cases, exceeding the 54% previously reported in feline cSCC (14). Literature regarding VEGF-A immunoexpression in feline cSCC is still scarce, and direct comparisons between studies are limited by differences in sample size, antibody clones, and immunohistochemical scoring systems employed. In dogs, VEGF-A has been more widely investigated, with positivity rates ranging from 32 to 100% (14, 46, 47).

Of the clinical variables evaluated in the present study, high VEGF-A immunoexpression was significantly associated with previous history of cSCC. In the present study, the majority of cats with a previous history of cSCC at presentation (6/9; 66.7%) had recurrent tumors, while three (33.3%) had new independent lesions at different sites of the head. The association between high VEGF-A expression and previous history of cSCC may partly reflect clonal selection, whereby surviving tumor cells acquire a progressively more aggressive and pro-angiogenic phenotype. Chronic UV-induced field cancerization may also contribute by upregulating VEGF-A expression in repeatedly damaged tissue, fostering a microenvironment permissive to tumor recurrence. However, these mechanisms remain speculative and warrant further investigation.

Interestingly, high VEGF-A expression was not significantly associated with subsequent tumor recurrence following ECT in the present study, in contrast to the significant association between VEGF-A expression and tumor recurrence previously reported in feline cSCC (14). Differences in treatment modality may have contributed to these findings, as the present study exclusively evaluated cats treated with ECT. Further investigations are warranted to determine whether the prognostic value of VEGF expression differs according to treatment approach.

In the present study, VEGF-A immunoexpression was also significantly associated with PNI. Beyond its role in brain vascularization, VEGF-A plays a key role in neuronal proliferation and survival (48). Reciprocally, nerve growth factor (NGF) and its receptor tyrosine kinase A (TrkA), key mediators of PNI, are able to stimulate endothelial cell migration and proliferation, contributing to the functional maturation of newly formed blood vessels (49, 50). These findings suggest that VEGF-driven angiogenesis and PNI may share common molecular mediators in feline cSCC, although the precise nature of this interaction warrants further investigation.

In the present study, VEGF-A immunoexpression was significantly associated with tumor invasion beyond the subcutaneous fat. However, initial evidence from human cSCC does not consistently support this association. A previous study demonstrated a significant increase in tumor vascular density exclusively in late-stage SCCs infiltrating the subcutaneous tissues, but without corresponding differences in VEGF-A immunoexpression across lesion groups (51). Similarly, another study found no significant correlation between VEGF-A expression and depth of tumor invasion (52). Despite these conflicting findings, we hypothesize that tumors invading deeper tissue compartments may experience greater hypoxia, a well-recognized inducer of VEGF-A upregulation, which could explain the higher VEGF-A immunoexpression observed in these cases. Further studies with larger cohorts are needed to elucidate this association in both feline and human cSCC.

To the authors’ knowledge, this is the first prospective study evaluating VEGF-A immunoexpression in association with overall survival time in cats with cSCC. No significant association was observed between VEGF-A expression and OST, DFI, or TTP. These findings are consistent with those reported by Dos Anjos et al. (47), who likewise found no significant association between VEGF immunoexpression and survival in dogs with cSCC treated with ECT. To date, no studies have evaluated the prognostic significance of VEGF-A expression in human cSCC treated with ECT, limiting direct comparisons with the present findings. In contrast, studies in human mucosal head and neck SCC treated with conventional therapeutic approaches have reported associations between high VEGF-A expression and poorer overall survival (53) as well as shorter disease-free intervals (54). These discrepancies may be related to the more aggressive biological behavior of mucosal SCC and to differences in treatment approaches compared with ECT-treated cutaneous tumors.

In the present study, expression of both EGFR-1 and VEGF-A was significantly associated with PNI. Although VEGF-A had no correlation with survival in the present study, these findings strengths the possible role of these biomarkers as prognostic indicators in feline cSCC. Beyond their potential prognostic value in this species, these biomarkers may also represent biologically relevant comparative biomarkers, further supporting the translational value of naturally occurring feline cSCC as a spontaneous model for human cutaneous squamous cell carcinoma.

Limitations of the present study include the absence of a normal skin control group for comparison, the relatively small sample size, and the inclusion of retrospective cases, which may have contributed to heterogeneity in data collection and follow-up completeness. Additionally, microvascular density was not assessed using endothelial markers such as CD31 or vWF. Therefore, it cannot be determined whether VEGF-A expression was associated with increased microvascular density or reflect functional tumor neoangiogenesis. EGFR-1 and VEGF-A expression was assessed exclusively by immunohistochemistry, without complementary validation using molecular techniques such as Western blotting or RT-qPCR. This lack of orthogonal validation should be considered when interpreting the semi-quantitative expression findings. Further prospective studies with larger cohorts are warranted to confirm our findings and to evaluate the predictive value of EGFR and VEGF-A as potential targets for inhibitor-based therapies in this species.

## Data Availability

The original contributions presented in the study are included in the article. Further inquiries can be directed to the corresponding author.
